# Draft Genome Sequence of the Intermediate Rough Vaccine Strain *Brucella abortus* S19Δ*per* Mutant

**DOI:** 10.1128/genomeA.01336-15

**Published:** 2015-11-12

**Authors:** Pallab Chaudhuri, Tapas K. Goswami T, Jonathan Lalsiamthara, Gurpreet Kaur, Udayakumar S. Vishnu, Jagadesan Sankarasubramanian, Paramasamy Gunasekaran, Jeyaprakash Rajendhran

**Affiliations:** a Division of Bacteriology & Mycology, Indian Veterinary Research Institute, Izatnagar, Uttar Pradesh, India; b Department of Genetics, School of Biological Sciences, Madurai Kamaraj University, Madurai, Tamil Nadu, India

## Abstract

Here, we report the genome sequence of the intermediate rough vaccine strain mutant, *Brucella abortus* S19Δ*per*. The length of the draft genome was 3,271,238 bp, with 57.2% G+C content. A total of 3,204 protein-coding genes and 56 RNA genes were predicted.

## GENOME ANNOUNCEMENT

Brucellosis is a zoonotic disease caused by *Brucella*. It infects a wide range of hosts. *Brucella abortus* strain S19 is widely used as the vaccine strain for the control of bovine brucellosis in different countries (1). Perosamine synthetase, coded by the *per* gene, polymerizes the monomeric units of *O*-polysaccharide (OPS) (2). This aids in establishing *Brucella* infection by inhibiting the fusion of lysosomes with phagosomes, which results in the formation of *Brucella*-containing vacuoles (3). A *B. abortus* S19Δ*per* mutant strain mounted a strong immune response with protective efficacy equivalent to or even better than that of the parent strain (*B. abortus* S19). The *B. abortus* S19Δ*per* mutant is more ideal than *B. abortus* S19 in terms of safety, potency, and differentiation of infected and vaccinated animals and is capable of serving as an alternate vaccine candidate for the control of bovine brucellosis (4). Here, we describe the draft genome sequence and annotation of the vaccine *B. abortus* S19Δ*per* mutant.

The *B. abortus* S19 *per* gene deletion plasmid pZper::kan was constructed by a backbone plasmid, pZErO-1 (Invitrogen, USA). The developed *per* gene deletion mutant of *B. abortus* S19 was named the *B. abortus* S19Δ*per* mutant (4). The genome was sequenced using the Ion Torrent personal genome machine (Life Technologies, Carlsbad, CA). In total, 1,135,644 reads with an average read length of 262 bp were obtained, which yielded 235 Mb of total sequenced bases with 71-fold coverage. The *de novo* assembly was performed using Mimicking Intelligent Read Assembly (MIRA) version 3.9.18 (5), which yielded 25 contigs. The largest contig was 444,937 bp long. The draft genome sequence of the *B. abortus* S19Δ*per* mutant was 3,271,238 bp long with 57.2% G+C content. The genome sequence was annotated using the Rapid Annotations using Subsystems Technology (RAST) server (6) and the NCBI Prokaryotic Genome Annotation Pipeline (http://www.ncbi.nlm.nih.gov/genome/annotation_prok/process/). The rRNA and tRNA genes were predicted using RNAmmer (7) and tRNAscan-SE 1.21 (8), respectively. A total of 3,260 genes were predicted, of which 3,204 are protein-coding genes. Overall, 2,584 of the protein-coding genes were assigned putative functions, and 620 genes were annotated as hypothetical proteins. A total of 56 RNA genes were predicted, of which 5 were rRNA genes and 51 were tRNA genes.

A genome-wide comparison showed 99.9% similarity with the complete genome of *B. abortus* S19 (accession numbers NC_010742 and NC_010740). A pairwise comparison revealed the deletion of the perosamine synthetase gene, *wbkB*, of chromosome one of *B. abortus* S19 by the insertion of a kanamycin resistance gene cassette (Kan^r^). In addition, >50 single-nucleotide polymorphisms (SNPs) were identified in the S19Δ*per* mutant strain.

### Nucleotide sequence accession numbers.

This whole-genome shotgun project has been deposited at DDBJ/EMBL/GenBank under the accession no. LFJE00000000. The version described in this paper is version LFJE01000000.
